# Artificial Intelligence-Assisted Detection of the Elongated Styloid Process on Dental Radiographic Images: A Systematic Review and Literature Update

**DOI:** 10.3390/jcm15134953

**Published:** 2026-06-25

**Authors:** Abdullah Alqarni, Hassan Ahmed Assiri, Ali Hassan Asiri, Sami Ali Humaidi, Hassan Abdulrhman Alshehri, Yousef S. Otayfi, Omar Saleh Aljughuli, Zaher Saleh Aljughuli, Abdulaziz Abdullah Alqahtani, Mohammad Shahul Hameed

**Affiliations:** 1 Department of Diagnostic Sciences and Oral Biology and Periodontology, College of Dentistry, King Khalid University, Abha 62521, Saudi Arabia; aawan@kku.edu.sa (A.A.);; 2 Department of Oral and Maxillofacial Radiology, College of Dentistry, Dental Hospital, Abha 61421, Saudi Arabia; 3 Internship Program, College of Dentistry, King Khalid University, Abha 62521, Saudi Arabiayousefaltyfy230@gmail.com (Y.S.O.);

**Keywords:** artificial intelligence, elongated styloid process, Eagle syndrome, stylohyoid ligament ossification, panoramic radiography, cone-beam computed tomography

## Abstract

**Background**: Elongated styloid processes and ossifications of the stylohyoid chain can be observed on dental imaging modalities. In this study, we assessed the performance of artificial intelligence (AI) in identifying elongated styloid processes and ossifications of the stylohyoid chain. **Methods**: We performed a systematic review of relevant studies published between April 2020 and April 2026 on PubMed, Scopus, and Web of Science. Relevant data were extracted using predefined criteria. We assessed the risk of bias using categories derived from QUADAS-2, CLAIM and STARD-AI. **Results**: Four original studies met the inclusion criteria. Of these, only two specifically addressed elongated styloid processes on panoramic images (OPGs). For one study that utilized ML algorithms, both logistic regression and neural networks achieved 100% performance, while naive Bayes demonstrated substantially lower performance than either model. Another study using deep learning algorithms observed accuracy rates of 97.49% and 84.11%, and area under the curve values of 0.9825 and 0.8943 for EfficientNetB5 and InceptionV3 models. A broader study using OPG anomaly detection reported target-level data for stylohyoid ligament ossification. The fourth study used cone-beam computed tomography images, including stylohyoid ligament ossification as part of a multi-class soft tissue calcification/ossification detection task. Due to significant variability in target definitions, imaging modalities, validation methods, and performance metrics across studies, a meta-analysis was not feasible. **Conclusions**: The use of AI-based systems for detecting elongated styloid processes and stylohyoid chain ossification shows potential for future clinical utility; however, current evidence is insufficient to support independent clinical practice. Future research should incorporate larger-scale prospective multicenter validations as well as external validation on a patient-by-patient basis when possible. Additional research into the clinical implications associated with both false-positive and false-negative results is warranted.

## 1. Introduction

The styloid process is an elongated bony projection that originates from the anterior aspect of the lateral wall of the external auditory meatus and forms the upper portion of the stylohyoid apparatus. It is connected to the hyoid bone via the stylohyoid ligament. Elongation of the styloid process or ossification within the stylohyoid chain may be identified as an incidental finding on various imaging modalities, including panoramic radiographs (OPG), cone-beam computed tomography (CBCT), computed tomography (CT), and CT angiography. An elongated styloid process identified radiographically should be distinguished from “Eagle Syndrome,” as many individuals with elongation or ossification of the stylohyoid structures remain asymptomatic; therefore, symptom-based clinical evaluation is necessary [1,2]. Despite advances in artificial intelligence (AI) for detecting and classifying elongated styloid processes, research in this area remains limited. There also exists variability in the scope of existing research. AI can potentially aid in locating elongated styloid processes or elongated stylohyoid chains, which can be missed during routine interpretation of OPGs focused primarily on dental concerns. This review aims to explore the effectiveness of AI for identifying and categorizing elongated styloid processes and stylohyoid chain ossifications from maxillofacial radiographic images, summarize related studies, and evaluate their quality and clinical applicability [1]. The styloid process is generally considered elongated when its measured length exceeds 30 mm [3]. The styloid process has also been evaluated in terms of morphology and ossification to determine the type of configuration present. One commonly used system is the Langlais classification, which categorizes radiographic appearance as elongated, pseudoarticulated, or segmented [4]. OPG is routinely used in dental practice to identify dental and jaw abnormalities. It offers the advantages of wide availability and lower cost when compared with advanced imaging modalities. However, it also has limitations that may affect diagnostic accuracy, such as superimposition of structures, magnification errors, and patient-positioning errors during image acquisition [5,6]. Compared with OPG, advanced imaging modalities such as CBCT and CT provide three-dimensional information, enabling a more accurate anatomical assessment of the styloid process. However, neither modality is generally justified for routine screening or the evaluation of incidental findings [4,5]. AI and deep learning (DL) methods are increasingly being adopted in dental and maxillofacial imaging. Convolutional neural networks have demonstrated high performance in detecting and classifying a wide range of dental and maxillofacial findings on OPG and CBCT images [6]. Recent studies have reported the successful application of AI in identifying dental anomalies, anatomical structures, pathologic lesions, and radiographic abnormalities, often achieving diagnostic performance comparable to that of experienced clinicians [7,8]. The increasing availability of digital imaging datasets, together with advances in computational methods, has further accelerated the integration of AI into dental radiology.

The use of AI in the radiologic identification and classification of elongated styloid processes has been studied less, as compared to its other applications. Several AI approaches have been employed in this task, including machine learning (ML) and DL. ML models learn patterns from data, often using hand-crafted features, whereas DL is a subset of ML that uses multilayer neural networks to learn features directly from images. Beyond image-based diagnostic systems, dental AI increasingly encompasses generative and large language model applications for clinical support, education, research writing, and patient communication [9]. This study specifically focuses on image-based AI for radiographic detection; generative AI represents a complementary and rapidly growing but distinct branch of dental AI [9].

The present study evaluates the accuracy of AI in detecting and classifying elongated styloid processes and stylohyoid chain ossifications from maxillofacial imaging, reviews prior research, and assesses the quality and clinical readiness of the available literature.

## 2. Materials and Methods

### 2.1. Study Protocol

**Figure 1:**
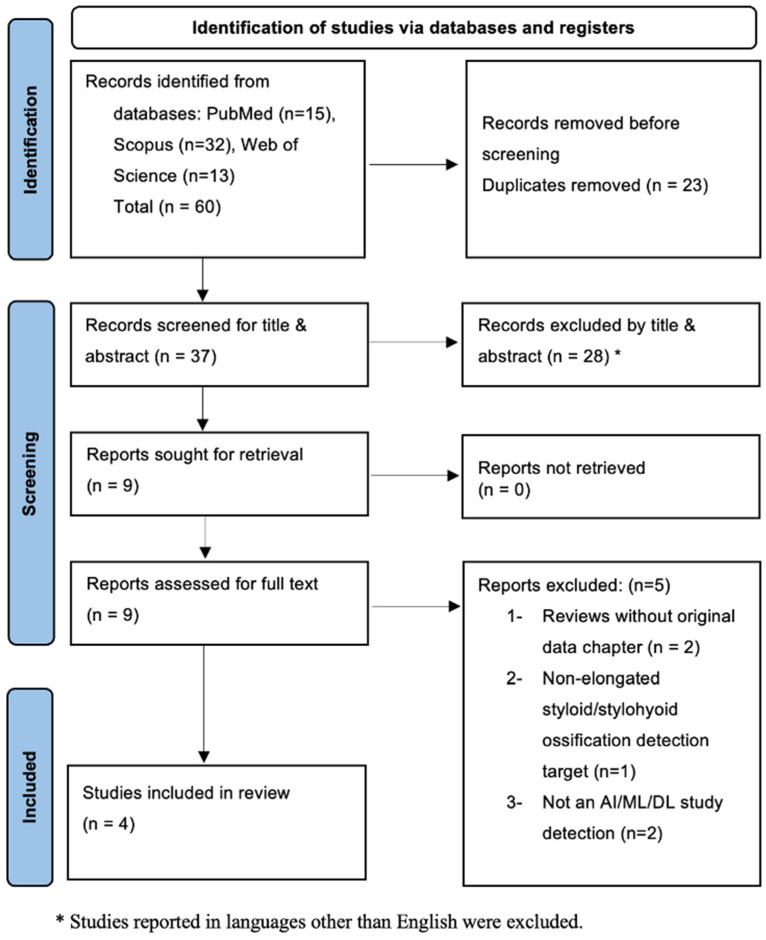
Study flow according to the PRISMA flowchart.

This study was conducted in accordance with the PRISMA 2020 guidelines (Figure 1) (Supplementary Materials). The study protocol was approved by the Scientific Research Committee of King Khalid University College of Dentistry under protocol number IRB/REG/2025/11. In addition, the protocol was registered in the INPLASY database under registration number INPLASY202650123; doi: 10.37766/inplasy2026.5.0123 available at https://inplasy.com/inplasy-2026-5-0123/ (accessed on 22 May 2026).

### 2.2. Research Question

The research question was framed as follows: “What is the diagnostic accuracy of AI in detecting elongated styloid processes?”.

### 2.3. Eligibility Criteria

The inclusion criteria comprised original studies that analyzed and reported the use of AI, ML, or DL to detect, classify, or localize one or more of the following targets: the elongated styloid process, ossification of the stylohyoid ligament, ossification within the stylohyoid chain, or soft-tissue calcification/ossification, including ossification of the stylohyoid ligament as a named subcategory. Eligible studies were required to report at least one performance metric, such as accuracy, sensitivity, specificity, precision, recall, F1-score, area under the curve (AUC), or target-specific counts. Studies were excluded if they were non-AI-based studies, anatomical case reports, reviews without original data, studies without evaluation of the stylohyoid or styloid structure as a target, or studies lacking sufficient information to extract performance metrics.

### 2.4. Search Strategy

A literature search was conducted in PubMed, Scopus, and Web of Science on 2 April 2026. Search terms combined styloid and stylohyoid concepts with AI- and imaging-related words. Only studies published in English were retrieved. The search strategy was designed to maximize sensitivity because the field was relatively small [10]. A summary of the database search strategies is shown in Table 1.

### 2.5. Data Extraction and Study Selection

Five reviewers independently screened the records using Rayyan^®^ (Rayyan Systems Inc., Cambridge, MA, USA); agreement at the full-text selection stage was high, and any disagreements were resolved by consensus, with a senior reviewer available to settle any unresolved cases. Given the small number of retrieved records, a formal kappa test was not calculated. Data were then extracted using a predefined, standardized data extraction table featuring author and year, country, study design, imaging modality, target condition, dataset, sample size, AI method, training and validation/split test, reference standard, observers, and external validation. Studies reporting only overall metrics for a broader task that included stylohyoid ligament ossification were treated as indirect evidence and not pooled with studies that directly evaluated elongated styloid processes.

### 2.6. Risk of Bias, Applicability, and Clinical Readiness

Risk of bias and applicability were assessed using the Quality Assessment of Diagnostic Accuracy Studies (QUADAS-2), which addresses patient or image selection, index testing, reference standards, and flow and timing. Risk-of-bias assessment was conducted by two independent authors and displayed using robvis, a tool designed to display the results of risk-of-bias assessments. Within the robvis output, the criteria “Some Concerns” is interpreted as indicating “unclear” risk-of-bias ratings in accordance with QUADAS-2 [11]. Each QUADAS-2 domain was independently rated by two reviewers as low, high, or unclear; a domain was rated “unclear” when reporting was insufficient to allow judgment, and each study’s overall risk of bias was assigned the “highest” rating across its four domains. CLAIM and STARD-AI were not used to modify these risk-of-bias judgments; they informed only the separate, descriptive clinical readiness indicators reported in Section 3.5 (external validation, patient-level independence, threshold reporting, reference-standard validity, transparency, and workflow relevance), which were tabulated narratively and not scored [12,13,14,15].

### 2.7. Data Synthesis

A narrative synthesis was conducted due to the substantial heterogeneity among the included studies, which precluded meaningful meta-analysis. Sources of heterogeneity included study objectives, imaging modalities, target definitions, AI architectures, validation approaches, and reported performance metrics. Greater emphasis was placed on studies that specifically evaluated elongated styloid processes compared with broader investigations on dental anomalies or soft-tissue calcifications.

## 3. Results

### 3.1. Study Identification

A total of sixty (60) records were retrieved from the database search. A duplicate check was performed using Rayyan^®^, yielding thirty-seven records suitable for title and abstract screening. Upon completion of a full-text review of nine records, only four studies met the inclusion criteria and were therefore included in the final analysis and quality assessment [16,17,18,19]. Conversely, five studies were excluded [20,21,22,23,24]; the specific reasons for exclusion are presented in Supplementary Table S1. The included studies were published between 2022 and 2026 and assessed AI applications for detecting elongated styloid processes and/or ossified stylohyoid ligaments in dental radiographic imaging.

### 3.2. Characteristics of Included Studies

As shown in Table 2, two of the four included studies directly addressed the detection and classification of elongated styloid processes using OPG and ML or DL approaches [17,18]. To avoid conflating evidence streams, the two studies that directly evaluated the elongated styloid process are presented separately from the two studies that addressed stylohyoid ligament ossification only within a broader detection framework, which constitutes indirect evidence [16,19]. The findings indicate that the dataset sizes ranged from a few hundred styloid images to approximately 23,000 OPG radiographs. A variety of AI approaches were used, including logistic regression, neural networks, EfficientNetB5, InceptionV3, and YOLOv5-based detection models. Although the findings demonstrated promising technological advances, heterogeneity was evident in imaging modalities, study populations, target definitions, and validation strategies.

### 3.3. AI Performance

The findings presented here should be interpreted as preliminary because only two of the four included studies directly evaluated elongated styloid process detection, and both were retrospective and internally validated. Jeevitha et al. [17] reported that logistic regression and neural network models achieved 100% accuracy, with no false-positives or false-negative results, whereas the naive Bayes model performed less favorably, achieving an AUC of approximately 0.782 and an accuracy of 0.715. The reported accuracy of 100% should be interpreted with caution rather than as evidence of clinical readiness; perfect performance on a small, internally validated dataset is a recognized warning sign of overfitting, data leakage, or image-level (rather than patient-level) data partitioning, and no independent or external validation was performed.

Ganesan et al. [18] reported that EfficientNetB5 achieved 97.49% accuracy, 98.00% precision, 97.00% recall, a 97.00% F1-score, and an AUC of 0.9825, whereas InceptionV3 achieved 84.11% accuracy and an AUC of 0.8943. The broader OPG anomaly detection study reported high sensitivity and specificity for ossification of the stylohyoid ligament but comparatively lower precision, suggesting a greater false-positive burden than may be acceptable for routine clinical deployment. The CBCT study on soft-tissue calcifications reported strong overall performance; however, styloid-specific performance metrics were not available from the accessible summary. A summary of AI performance for the relevant studies is presented in Table 3.

### 3.4. Risk of Bias Assessment and Applicability

**Figure 2:**
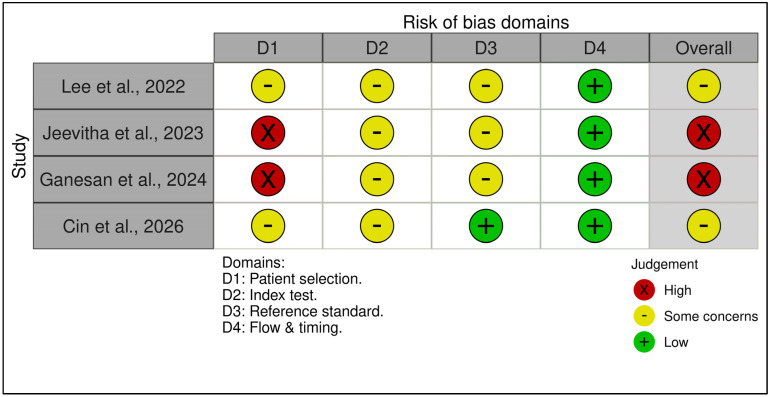
Risk-of-bias traffic light plot for the included studies (QUADAS-2), generated using robvis. D1 refers to patient selection; D2 to index test; D3 to reference standard; D4 to flow and timing. “Green +”: Low risk of bias. Yellow “−”: Unclear risk of bias (“Some concerns” in the robvis Legend). Red “×”: High risk of bias. The overall column shows the **worst (highest)** risk-of-bias rating across the four domains (worst-domain rule). Separate applicability concerns are found in Table 4 [16,17,18,19].

**Figure 3:**
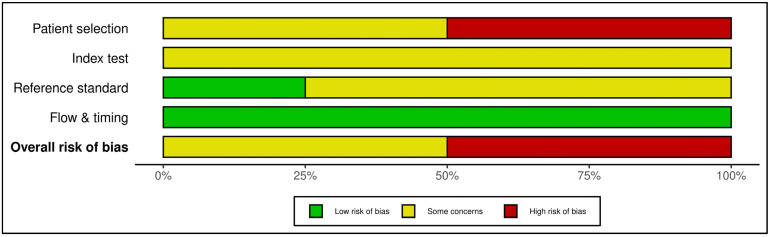
The QUADAS-2 “risk-of-bias” traffic light plots were produced using the software robvis. D1 represents Patient Selection; D2, Index Test; D3, Reference Standard; D4, Flow and Timing. Green “+” is a low risk of bias. Yellow “−” is an uncertain risk of bias (which was displayed as “Some Concerns” on the robvis legend). Red “×” is a high risk of bias. The Overall column shows the **worst (highest)** risk-of-bias rating across the four domains (worst-domain rule). Applicability issues are listed separately in Table 4.

Overall, the certainty of the evidence was low, as indicated by the risk-of-bias assessment (Table 4), largely because of incomplete reporting. The primary recurring concerns were retrospective image selection, the use of curated image datasets rather than consecutive clinical populations, possible image-level rather than patient-level data splitting, limited or absent external validation, and reliance on OPG radiographic measurements as the reference standard. The flow and timing domain was assessed as low risk of bias for all included studies since each study evaluated data retrospectively from a single dataset in which each image was assessed using both the index test and reference standard. Therefore, there were no issues related to time intervals or partial verification. The results at both the domain level and the overall level are presented using a traffic light plot (Figure 2), which illustrates each study’s overall quality, and in a bar chart (Figure 3), which summarizes the included studies. Patient selection was regarded as high risk of bias in two of the four studies (50%) and unclear in the other two (50%); the high-risk ratings corresponded to the two direct elongated-styloid-process studies, which used curated, non-consecutive image sets rather than consecutive clinical samples. In contrast, the reference standard was judged low risk in one study (25%) and unclear in three (75%). “In summary, all four studies assessed the flow and timing of evaluations and found that they were all at “low risk of bias.” Therefore, two studies were classified as having a “high risk of bias,” and the remaining two studies were classified overall as “unclear”; none of the studies were classified as having a “low risk of bias.” Applicability varied across the included studies. Two studies directly evaluated AI models for detecting elongated styloid processes and were therefore closely aligned with the review objective. In contrast, the remaining two studies assessed stylohyoid ligament ossification within broader frameworks for detecting dental anomalies or soft-tissue calcifications, making their relevance more indirect. In addition, all studies relied on single-center or curated datasets and lacked external validation, which may limit the generalizability of the findings.

### 3.5. Clinical Readiness

Although several promising performance metrics were reported, the overall clinical readiness of current AI to detect elongated styloid processes is low. There were no studies employing prospective multicenter external validation. Most studies relied on OPG measurements or manual labeling as their reference standard (Table 5). Additionally, inconsistency was observed among studies with respect to patient-level independence. In general, predefined clinical decision thresholds were largely absent. Furthermore, none of the studies investigated how AI would affect clinical workflow, diagnostic efficiency, or patient outcomes. Additionally, limited access to source code and external validation datasets restricts both the reproducibility of these findings and the ability to independently validate the reported results.

## 4. Discussion

This study identified growing evidence on the use of AI to detect and classify elongated styloid processes and stylohyoid chain ossifications on OPG and CBCT. Although AI applications demonstrated high performance in identifying these findings, the small number of available studies and the substantial heterogeneity among them limit meaningful pooled diagnostic accuracy estimates. These limitations also restrict the ability to recommend AI systems for routine clinical use without further validation. The most directly relevant studies were the ML study by Jeevitha et al. and the DL classification study by Ganesan et al. [17,18]. Both demonstrated high performance, although their findings should be considered proof-of-concept evidence rather than definitive evidence of diagnostic accuracy.

However, obtaining acceptable performance on small retrospectively collected datasets does not always indicate that there will not be an issue with “data leakage,” where one or more of the above-mentioned issues exist [17,18]. The large-sample study reported by Lee et al. was considered clinically informative since it evaluated multiple dental and maxillofacial anomalies on routine OPG and included stylohyoid ligament ossification as a target [16]. The stylohyoid ligament class demonstrated high sensitivity and specificity but only modest precision. This pattern is potentially acceptable in a triage setting where the primary goal is to minimize unidentified findings; however, it may also create a substantial review burden if implemented without appropriate user-interface design or confidence thresholds. The CBCT study conducted by Cin et al. is also noteworthy, as CBCT provides a three-dimensional assessment and overcomes some of the limitations of OPG imaging [19]. However, this study investigated soft-tissue calcifications and ossifications as a broader category, and the available reports did not provide styloid-specific metrics. Therefore, it should be considered supportive evidence that AI can detect calcified or ossified structures on CBCT rather than direct evidence of accurate elongated styloid process detection. A key methodological consideration is the reference standard. A CBCT-referenced study of OPG diagnosis reported good specificity but low sensitivity for styloid process elongation, with sensitivities of 67.3% and 64.4% on the right and left sides, respectively [5]. This raises an important concern: if an AI model is trained and tested solely on manually labeled OPGs, it may reproduce the limitations of OPG imaging rather than accurately reflect anatomical status. Similar concerns have been highlighted in a systematic review, and CBCT has been recommended as the preferred imaging modality because it enables three-dimensional assessment of styloid process length and morphology without superimposition [25]. When the primary focus is on aspects such as length, angulation, closeness to vascular elements, or surgical planning for a patient’s clinical use, CBCT or CT-based reference standards are the best options. There is significant variability in how elongation of the styloid process has been defined, measured, and classified across studies. Due to the large amount of anatomical variability (length, shape, segmentation, and mode of ossification) associated with the styloid process, different thresholds have been applied across studies to define elongation [26,27]. The use of the term “Eagle syndrome” should be used cautiously. An elongated styloid process detected using AI is not synonymous with Eagle syndrome; the finding can occur in asymptomatic patients who have radiographically elongated processes. Additionally, as the classification of Eagle syndrome has evolved into discrete clinical syndromes, it demonstrates that establishing standardized criteria for the diagnosis of this entity remains difficult [28]. Consequently, a clinically useful AI tool should be positioned as an imaging aid that identifies elongation or stylohyoid chain ossification and supports structured reporting. It should not be used as a standalone diagnostic tool for Eagle syndrome without consideration of clinical history, physical examination findings, and appropriate radiologic correlation [29]. All four categories discussed above (binary detection, multi-class morphology classification, wide-spectrum anomaly detection, and CBCT-based soft-tissue calcification detection) vary significantly by denominator, definition of targets, operating threshold, and clinical relevance. As such, pooling these would likely yield a statistically correct summary effect estimate, yet one that could be practically misleading. The most significant finding from this review is the identification of an obvious “evidence gap” in the field. Many new studies are reporting high performance on curated datasets; however, progress in the field requires rigorous testing/validation of image-analysis systems in real-world clinical environments. Future research should validate locked models across multiple centers using consecutive, patient-level OPG datasets; account for the dependence introduced by bilateral structures from the same patient; and compare AI-assisted with unaided clinicians’ performance, including the clinical impact of false-positive and false-negative results.

This systematic review has several limitations. First, our search was limited to PubMed, Scopus, and Web of Science; although these provide broad coverage of biomedical and dental imaging literature, records indexed in engineering-oriented databases such as IEEE Xplore, Embase, or the ACM Digital Library may have been missed. The deliberately sensitive search strategy was designed to reduce the likelihood of omitting eligible studies. Furthermore, the current evidence base regarding the performance of AI is very small. Only two studies used AI to assess elongated styloid processes. Some studies that may have been pertinent to this systematic review were only presented as abstracts or publisher summaries. Therefore, we were unable to extract specific data related to elongated styloid processes from these sources.

## 5. Conclusions

AI-assisted detection and classification of elongated styloid processes and stylohyoid chain ossifications demonstrate promising results, and direct studies using OPG reported high accuracy. However, current evidence relies on retrospective, curated, and predominantly internally validated datasets; therefore, it supports only preliminary research utility and does not yet justify clinical deployment. Future studies should use consecutive, patient-level datasets with prospective multicenter external validation, standardized definitions of styloid process elongation and stylohyoid ossification, CBCT- or CT-based reference standards when anatomical accuracy is required, predefined clinical decision thresholds linked to reporting or referral pathways, and reporting aligned with STARD-AI and CLAIM.

## Figures and Tables

**Table 1:** Search strategy used to identify AI studies on elongated styloid processes or stylohyoid chain ossifications.

Source	Search Strategy or Search Concept
PubMed	(styloid*[tiab] OR stylohyoid*[tiab] OR “Eagle syndrome”[tiab] OR “Eagle’s syndrome”[tiab]) AND (“Artificial Intelligence”[Mesh] OR “artificial intelligence”[tiab] OR “machine learning”[tiab] OR “deep learning”[tiab] OR “neural network”[tiab] OR “neural networks”[tiab] OR convolutional[tiab] OR CNN[tiab] OR “computer-aided”[tiab] OR “computer aided”[tiab] OR “automated detection”[tiab] OR “automatic detection”[tiab] OR “object detection”[tiab] OR “computer vision”[tiab] OR radiomics[tiab] OR YOLO[tiab])
Scopus	TITLE-ABS-KEY ((styloid* OR stylohyoid* OR “Eagle syndrome”) AND (“artificial intelligence” OR “machine learning” OR “deep learning” OR “neural network*” OR “convolutional neural network*” OR cnn OR “computer-aided” OR “computer vision” OR “object detection” OR radiomics OR yolo OR “automated detection” OR “automatic detection” OR “deep neural network*”))
Web of Science	TS = ((styloid* OR stylohyoid* OR “Eagle syndrome”) AND (“artificial intelligence” OR “machine learning” OR “deep learning” OR “neural network*” OR “convolutional neural network*” OR CNN OR “computer-aided” OR “computer vision” OR “object detection” OR radiomics OR YOLO OR “automated detection” OR “automatic detection” OR “deep neural network*”))

**Table 2:** Data extraction of relevant studies.

Author and Year	Country	Study Design	Imaging Modality	Target Condition	Dataset	Sample Size	AI Method	Training and Validation/Test Split	Reference Standard	Observers	External Validation
Lee et al., 2022 [16]	Republic of Korea	Retrospective study; a deep-learning object-detection model trained to recognize 17 dental and maxillofacial anomalies on OPG radiographs	OPG	Stylohyoid ligament ossification, assessed as one of 17 anomalies (indirect evidence)	Pragmatic, expert-labeled archive of 22,999 OPG radiographs from 30 dental clinics (July 2020–July 2021)	22,999 OPG radiographs (the stylohyoid test set contained 157 positive findings and 1275 negative images)	Object-detection pipeline based on deep learning	Chronological split, partitioned at the image level: July 2020–March 2021 for training and validation, April–July 2021 for testing (percentages not reported)	Manual annotation on the radiograph (bounding box refined to a polygon), a detection counted correct when overlap with the reference exceeded 50%	One (a single dental-radiology expert)	No—the test set came from the same 30 clinics
Jeevitha et al., 2023 [17]	India	Retrospective single-center diagnostic study; three machine-learning classifiers distinguished elongated from normal styloid processes	OPG	Elongated styloid process (direct evidence)	400 OPG radiographs screened and curated to 369 cropped images (169 elongated, 200 normal), all acquired on one machine (Carestream CS8100SC)	369 cropped OPGs (from 400 screened)	Logistic regression, neural network, and naive Bayes using the Orange software	Half the images for training and half for testing, with cross-validation and no separate validation set; partitioned at the image level, with bilateral cases cropped into two images	A single calibrated observer measured styloid length in ImageJ, classifying processes longer than 30 mm as elongated	One (a single calibrated observer)	No—single center with internal cross-validation
Ganesan et al., 2025 [18]	India (images from SRM Dental College, Chennai; one co-author affiliated with Ajman University, United Arab Emirates)	Retrospective study comparing two deep-learning image-classification models (EfficientNetB5 and InceptionV3), with morphology typed by the Langlais classification	OPG	Elongated styloid process (direct evidence)	938 archived OPG radiographs screened and curated to 450 images (330 elongated, 120 normal)	450 OPGs (330 elongated, 120 normal; from 938 screened)	Efficient NetB5 and InceptionV3	90 images (20%) held out for testing, and the remaining 360 (80%) used for training and validation; partitioned at the image level	A single calibrated observer measured styloid length in ImageJ (elongation defined as >30 mm) and typed morphology using the Langlais classification	One (a single calibrated observer)	No—the held-out test set came from the same archive
Cin et al., 2026 [19]	Turkey	Retrospective study; a deep-learning model detected soft-tissue calcifications and ossifications on CBCT in single-class and multi-class settings	CBCT	Styloid (stylohyoid) ligament ossification, assessed as one of about ten calcification/ossification classes (indirect evidence)	CBCT scans from 287 patients reviewed retrospectively, with calcifications identified across axial, coronal and sagittal planes and segmented in the axial plane	287 patients (CBCT)	YOLOv5-based detection model	80% training, 10% validation and 10% testing (split unit not stated in the available abstract)	Expert identification of soft-tissue calcifications and ossifications with axial-plane segmentation on CBCT	Not reported in the available abstract	No—internal 80/10/10 split

**Table 3:** AI performance metrics and clinical interpretation.

Study	AI Model(s)	Validation Type	Reported Performance	Interpretation
Lee et al., 2022 [16]	Faster R-CNN object detector (with Detectron2 post-processing)	Internal, using a chronologically held-out test set	For stylohyoid ligament ossification, precision was 56.4%, sensitivity was 95.5%, and specificity was 96.8%	Detection was sensitive and specific but imprecise; the target was only one of 17 classes (indirect evidence), and no external validation was performed
Jeevitha et al., 2023 [17]	Logistic regression, neural network and naïve Bayes (Orange software)	Internal, using a 50/50 split with cross-validation	Logistic regression and the neural network reached 1.00 on accuracy, precision, recall, F1 and AUC; naïve Bayes was weaker (AUC 0.782, accuracy 0.715, F1 0.682, precision 0.697, recall 0.668)	The perfect scores came from a small, internally validated dataset and should be read cautiously, as they are a recognized signal of overfitting or data leakage; no external validation was performed
Ganesan et al., 2025 [18]	EfficientNetB5 and InceptionV3	Internal, using a held-out 20% test set	EfficientNetB5 reached 97.49% accuracy (precision 98%, recall 97%, F1 97%, AUC 0.9825); InceptionV3 reached 84.11% accuracy (precision 85%, recall 84%, F1 84%, AUC 0.8943)	Both models performed well on a curated dataset, but validation was limited to an internal test set with no external validation
Cin et al., 2026 [19]	Not named in the available abstract (the manuscript cites YOLOv5—to be confirmed)	Internal, using an 80/10/10 split	The single-class model reached a sensitivity of 0.98, precision of 0.91 and F1 of 0.94; for the same variables, the multi-class model reached values of 0.88, 0.80 and 0.84, respectively. These are overall figures, with no styloid-specific metrics reported.	Evidence is indirect, with no styloid-specific accuracy; the CBCT reference standard is a strength, but no external validation was performed

CBCT, cone-beam computed tomography; AUC, area under the curve.

**Table 4:** Quality assessment of diagnostic accuracy studies (QUADAS-2)-informed risk-of-bias and applicability summary.

Study	Patient/Image Selection	Index Test	Reference Standard	Flow/Timing	Overall RoB	Applicability
Lee et al., 2022 [16]	Unclear	Unclear	Unclear	Low	Unclear	Indirect: stylohyoid ligament ossification evaluated within a broader dental anomaly detection framework
Jeevitha et al., 2023 [17]	High	Unclear	Unclear	Low	High	Direct target; however, the study was single-center and internally validated
Ganesan et al., 2025 [18]	High	Unclear	Unclear	Low	High	Direct target; however, the study used a curated image-level dataset and lacked multicenter external validation
Cin et al., 2026 [19]	Unclear	Unclear	Low	Low	Unclear	Indirect: CBCT STCO model included stylohyoid ligament ossification, but only overall metrics were available

CBCT, cone-beam computed tomography; STCO, soft tissue calcification and ossification.

**Table 5:** Clinical readiness of AI for elongated styloid process and stylohyoid chain ossification detection.

Domain	Current Evidence	Implication
External validation	No dedicated elongated styloid process model has been prospectively validated across multiple external centers.	Performance may be inflated by local image-acquisition protocols and dataset curation.
Reference standard	Most evidence relating to elongated styloid processes relied on OPG measurements or manual labels.	CBCT-referenced validation is needed when precise length and morphology are clinically important.
Patient-level independence	The unit of analysis was often an image or a cropped styloid process.	Bilateral structures from the same patient may introduce dependence if not appropriately addressed.
Clinical threshold	Most studies did not prespecify a clinical decision threshold linked to referral, CBCT imaging, or symptom evaluation.	Reported accuracy metrics are difficult to translate into clinical workflows.
Outcome relevance	No study evaluated the effect of AI on radiologists’ or dentists’ efficiency, missed incidental findings, false-positive workload, or patient outcomes.	Readiness for clinical implementation remains limited.
Transparency	Source code and external test datasets were generally unavailable.	Independent replication and validation remain difficult.

CBCT, cone-beam computed tomography.

## Data Availability

All data are contained within the article or Supplementary Material.

## Supplementary Materials

The following supporting information can be downloaded at: https://www.mdpi.com/article/10.3390/jcm15134953/s1. RISMA_2020_checklist; Table S1. Reports excluded at full-text assessment, with reasons (n = 5).
