# Supplementary material for: Artificial Intelligence-Assisted Detection of the Elongated Styloid Process on Dental Radiographic Images: A Systematic Review and Literature Update
Source: J Clin Med. 2026 Jun 25;15(13):4953. doi: 10.3390/jcm15134953 (PMC13361565; doi:10.3390/jcm15134953)
Supplement: Supplementary file 1 [file jcm-15-04953-s001.zip › jcm-4398363-supplementary -1.pdf]

Supplementary Table S1. Reports excluded at full-text assessment, with reasons (n = 5).

No.	Excluded report	Reason for exclusion (criterion in Section 2.3)
20	Cammaroto G, Migliorelli A, Pelucchi S, Meccariello G, De Vito A. Social (Chats), Websites, and ChatGPT in Eagle Syndrome. In: Vicini C, Zamboni P, Iannella G, editors. EAGLE Syndrome: The Many Faces of the Elongated Styloid Process. Cham: Springer Nature Switzerland; 2025. p. 33–42.	Review without original data (book chapter; no original AI performance data).
21	Browning J, Kornreich M, Chow A, Pawar J, Zhang L, Herzog R, et al. Uncertainty-Aware Deep Reinforcement Learning for Anatomical Landmark Detection in Medical Images. In: Medical Image Computing and Computer Assisted Intervention – MICCAI 2021. Cham: Springer; 2021. p. 636–44.	Did not evaluate the styloid/stylohyoid process as a target (AI method for general anatomical landmark detection).
22	Dou G, Zhang Y, Zong CL, Chen YL, Guo YX, Tian L. Application of surgical navigation in styloidectomy for treating Eagle's syndrome. Ther Clin Risk Manag. 2016;12:575–83.	Not an AI/ML/DL study (surgical-navigation/treatment study).
23	Campisi R, Vicini C, Caranti A, De Carli F, Visani P, Monetti G. Eagle Imaging 2. In: EAGLE Syndrome: The Many Faces of the Elongated Styloid Process. Cham: Springer; 2025. p. 87–100.	Review without original data (book chapter; narrative imaging overview).
24	Tiburtino V, Filho G, Gabriel L, Andrade B, De P, Cruz S, et al. Prevalence of Elongated Styloid Process in 1,000 Panoramic Radiographs of a Brazilian Population. European Journal of Medical and Health Sciences. 2024;6:35.	Not an AI/ML/DL study (manual prevalence survey).

Reasons correspond to the eligibility criteria pre-specified in Section 2.3. Numbering matches the reference list ([20]–[24]).
